# Cross-Border Reproductive Care: Psychological Distress in A Sample of Women Undergoing *In Vitro* Fertilization Treatment with and without Oocyte Donation

**DOI:** 10.22074/ijfs.2020.5997

**Published:** 2020-07-15

**Authors:** Gracia Lasheras, Gemma Mestre-Bach, Elisabet Clua, Ignacio Rodríguez., Borja Farré-Sender

**Affiliations:** 1 Department of Psychiatry, Psychology and Psychosomatics, Dexeus University Hospital, Barcelona, Spain; 2 Facultad de Ciencias de la Salud. Universidad Internacional de La Rioja, La Rioja, Spain; 3 Department of Obstetrics, Gynaecology and Reproduction, Dexeus University Hospital, Barcelona, Spain

**Keywords:** Assisted Reproductive Technologies, *In Vitro* Fertilization, Oocyte Donation, Psychopathology, Personality

## Abstract

**Background:**

Cross-border reproductive care (CBRC) refers to the movement of patients to foreign countries for fertility treatment. Limited evidence indicates that this phenomenon is associated with a risk of psychologi- cal distress, but few studies on the psychological impact of CBRC are currently available. The aim of this study was to compare the anxiety and depression levels of a group of cross-border patients with a local Spanish patient group, both of which underwent *in vitro* fertilization (IVF) treatment. We also sought to explore the clinical, sociodemographic and personality profiles of the CBRC group and local women.

**Materials and Methods:**

This present cross-sectional study was conducted on 161 infertile females (71 CBRC patients and 90 local women) who were undergoing IVF treatment. The following questionnaires were used to collect data: Spielberger State Anxiety Inventory (STAI-S), the Beck Depression Inventory-II (BDI-II) and the Zuckerman-Kuhlman Personality Questionnaire (ZKPQ). Sociodemographic, clinical, reproductive and CBRC variables were also recorded.

**Results:**

CBRC patients, specifically CBRC oocyte recipients, showed higher levels of anxiety compared to lo- cal women. However, no significant differences in depression scores were found between both groups. Finally, when analysing personality, the Activity scale scores of the ZKPQ were found to be higher in CBRC oocyte recipients, which indicated a greater tendency for general activity and higher energy levels.

**Conclusion:**

CBRC oocyte recipient women may have greater vulnerability to anxiety than local women prior to infertility treatment. Screening and psychological support protocols for anxiety in this population should be considered.

## Introduction

Infertility is defined as the failure to establish a clinical pregnancy after 12 months of regular, unprotected sexual intercourse. The prevalence of infertility is between 17% and 28% in industrialized countries (1). Cross-border reproductive care (CBRC), also known as reproductive tourism, refers to a phenomenon in which infertile patients travel to other countries to obtain specific assisted reproduction treatments (ART), mainly intrauterine insemination, *in vitro* fertilization (IVF) and oocyte donation (2). Several clinical, social and legal issues can make the treatment more difficult in the patients’ country of origin, which leads patients to seek CBRC. In most cases, the treatment is forbidden by law in the country of origin for the following reasons: it is considered an unsafe technique (e.g., oocyte freezing); ethical considerations (e.g., gamete donation, preimplantation genetic diagnosis), or patient characteristics (e.g., postmenopausal or homosexual women). Likewise, excessively long waiting lists in one’s home country, lack of expertise (e.g., preimplantation genetic diagnosis) and high financial costs also block access to reproductive treatments (3). Consequently, there has been a progressive increase in migration to obtain ART. With regards to Europe, countries in which CBRC is most prevalent are France, Italy and Germany, 79% of those applicants are treated in Spain mostly due to the absence of legal restrictions in gamete donation. In 2016, the Spanish Fertility Society (SEF) Registry documented 12 939 ART cycles. The majority of these patients were from Italy and France. A total of 86% of cases had gamete donation, which was mostly from an oocyte source (53.3%) (4).

Infertility can have a negative impact on quality of life (5- 7), and the IVF treatment process is associated with stress (8), depression, anxiety and psychosomatic symptoms that interfere with fertility treatment (9). In terms of personality traits, some studies have found that 'sensation seeking' profiles are less prevalent in infertile women, who tend to engage in more experiential avoidance and self-judgment coping mechanisms (10). Evidence suggests that neuroticism, the relatively stable tendency to respond with negative emotions (hostility, sadness, anxiety, etc.) to threat, frustration, or loss (11) is related to the presence and maintenance of anxiety and depressive symptoms/disorders (12). It remains unclear, however, if the same holds true in infertile women and in women who seek CBRC.

In women who seek CBRC, it appears that multiple elements combine to increase vulnerability to psychopathological conditions. According to data from the SEF Registry, more than half of CBRC women in Spain seek oocyte donation. This, in turn, entails resorting to what may be considered as illegal fertility techniques in their respective countries (13). In addition, these women are usually older; have a longer duration of infertility; experience an increased conception failure rate; and have more difficulties in attaining healthy and normal births (14). In addition, further stress results from living in and adapting to unfamiliar environments, seeking justification for their work absence, the economic expense of treatment (15), and the travel costs involved.

Numerous studies carried out from a medical-legal perspective mainly considered the ethical aspects of this practice (16,17). However, only some studies have highlighted the fact that the adaptation to a different medical care context together with the associated psychological/ economic discomfort caused by displacement can interfere with the quality of life of these patients. Therefore, studies relating CBRC to psychopathological consequences are needed.

In order to improve treatment interventions for patients who receive CBRC, a better understanding of the mechanisms underpinning its associated psychiatric symptomatology is required. To our knowledge, no empirical study has yet explored the association between CBRC and psychopathology in women. As such, we aimed to determine if there were differences in emotional states and personality profiles of women who receive CBRC in comparison to local women. Our study aims were twofold: a) to compare anxiety and depression levels between the CBRC patient group and the local patient group and b) to explore the sociodemographic, clinical and personality profiles of both groups. We hypothesized that CBRC patients: 1. would show higher anxiety and depression levels derived from factors associated with displacement and 2. would show a distinct personality profile in comparison with local women.

## Materials and Methods

### Sample

The present cross-sectional study was conducted at a hospital in Barcelona, Spain between October, 2015 and March, 2016. A total of 163 women were recruited through the Department of Reproductive Medicine in a hospital of Barcelona (Spain) at the beginning of IVF treatment with either their own or donated oocytes through convenience sampling, so that the samples were selected based on availability. The local group comprised 90 local Spanish women who underwent IVF treatment. Initially, the CBRC group was comprised of 73 women from other countries who sought IVF treatment in Spain; however, only two women were not Italian. In order to homogenize the sample, these two non-Italian women were excluded.

Inclusion criteria for both groups were: infertile female aged between 18 and 50 years, need for IVF treatment with or without oocyte donation, completed primary school as the minimum level of education, agreed to participate in the study, and signed the informed consent. The local group included women from Spain, while the CBRC group only included women from other countries initially, and finally just Italy, who travelled to Spain for IVF treatment.

Exclusion criteria in the local group was: an insufficient level of Spanish needed to complete the self-administered questionnaires and, in the CBRC group, an insufficient level of Italian needed to complete the self-administered questionnaires.

### Procedure

Comprehensive clinical and psychological evaluations were carried out the week prior to the transfer along with the collection of additional reproductive, clinical and demographic data. The week prior to the transfer was considered a homogeneous moment for all patients and was not influenced by ongoing treatment variables, nor did it interfere with the CBRC group's return to Italy. Two staff biologists from the Reproductive Medicine Department in our hospital explained the basis of the study to the participants and, if they agreed to participate, they were required to sign the informed consent forms. At the gynaecology offices, staff asked the patients to complete four study questionnaires - the Spielberger State Anxiety Inventory (STAI-S), Beck Depression Inventory-II (BDI-II), Zuckerman-Kuhlman Personality Questionnaire (ZKPQ), and a socio-demographic, clinical and reproductive questionnaire.

### Instruments

#### Spielberger State Anxiety Inventory

This 20-item questionnaire was used to assess the current mood of the respondent. All items were rated on a 4-point scale, from “Almost Never” 1. to “Almost Always” 2. which resulted in total scores from 20 to 80, with higher scores indicative of greater levels of anxiety. Internal consistency coefficients for the original scale ranged from 0.86 to 0.95, whilst test-retest reliability coefficients ranged from 0.65 to 0.75 over a two month interval. The STAI-S, a widely used sub-scale, was the only variable from this questionnaire used in the present study. Two different validated translations were used for each sample population, Spanish (18) and Italian.

### Beck Depression Inventory-II

The beck depression inventory-II (BDI-II) is an instrument for rating the severity of depressive symptoms. The BDI-II contains 21 items with four statements rated on a 0-3 scale from “Almost Never” to “Almost Always”, and a total score from 0 to 63. This instrument categorizes depression using a low 14-19, moderate 20-28, or severe 29-63 stratum. Internal consistency for the original BDI scale ranges from 0.73 to 0.92 with a mean of 0.86 (19). Two different validated translations were used for each sample population of the study: Spanish and Italian (20).

### Zuckerman-Kuhlman Personality Questionnaire

This 99-item questionnaire has a true/false format and assesses personality traits according to five personality factors: Neuroticism-Anxiety (19 items), Activity (17 items), Sociability (17 items), Impulsive Sensation Seeking (19 items), and Aggression-Hostility (17 items). Additionally, it has an Honesty scale (10 items) in order to ensure the reliability of the results. The original version features favourable psychometric properties of a high internal consistency (Cronbach’s alpha range: 0.77 to 0.91), in addition to satisfactory convergent, discriminant, and consensual validity (21). Two different validated translations were used for the study: Spanish (22) and Italian (23).

### Socio-demographic, clinical and reproductive variables interview

Additional clinical, demographic and reproductive variables were measured via a self-administered structured questionnaire created socio-demographic, clinical and reproductive variables interview (ad hoc) for this study. We included clinical and demographic variables of age, community origin, partner gender, education level, occupation and psychiatric history. In addition, the questionnaire explored cross-border issues such as: causes of movement; companions; perceived psychological discomfort; relevant difficulties in cross-border infertility treatment; and an evaluation of help received from language facilitation institutions during the process. Reproductive history was also taken into account, e.g., quantifying the number of living children; duration of infertility, previous failure(s) with assisted reproduction technology cycles (intrauterine insemination, IVF, oocyte donation); and previous miscarriages.

### Ethics

The study was carried out in accordance with the latest version of the Declaration of Helsinki. Signed informed consent was obtained from all participants, and approval was granted from the Hospital Institutional Review Board.

### Statistical analysis

All statistical analysis was performed using IBM SPSS Statistics for Windows, version 20.0 (IBM Corp., Armonk, NY, USA). Comparison between categorical variables was carried out using the chi-square tests (χ2) and the t-test. All tests were bilateral with a significance level set to α=0.05.

## Results

### Descriptive for the sample

Table 1 shows participants’ descriptives at intake (baseline values) and a comparison between the CBRC and local patients. Both groups had similar sociodemographic characteristics and no observed significant differences. No statistical differences were found in personal psychiatric history between the groups except for a higher than average incidence of previous IVF with the patients’ own oocytes in the CBRC group.

### Cross-border reproductive care issues

**Fig 1:**
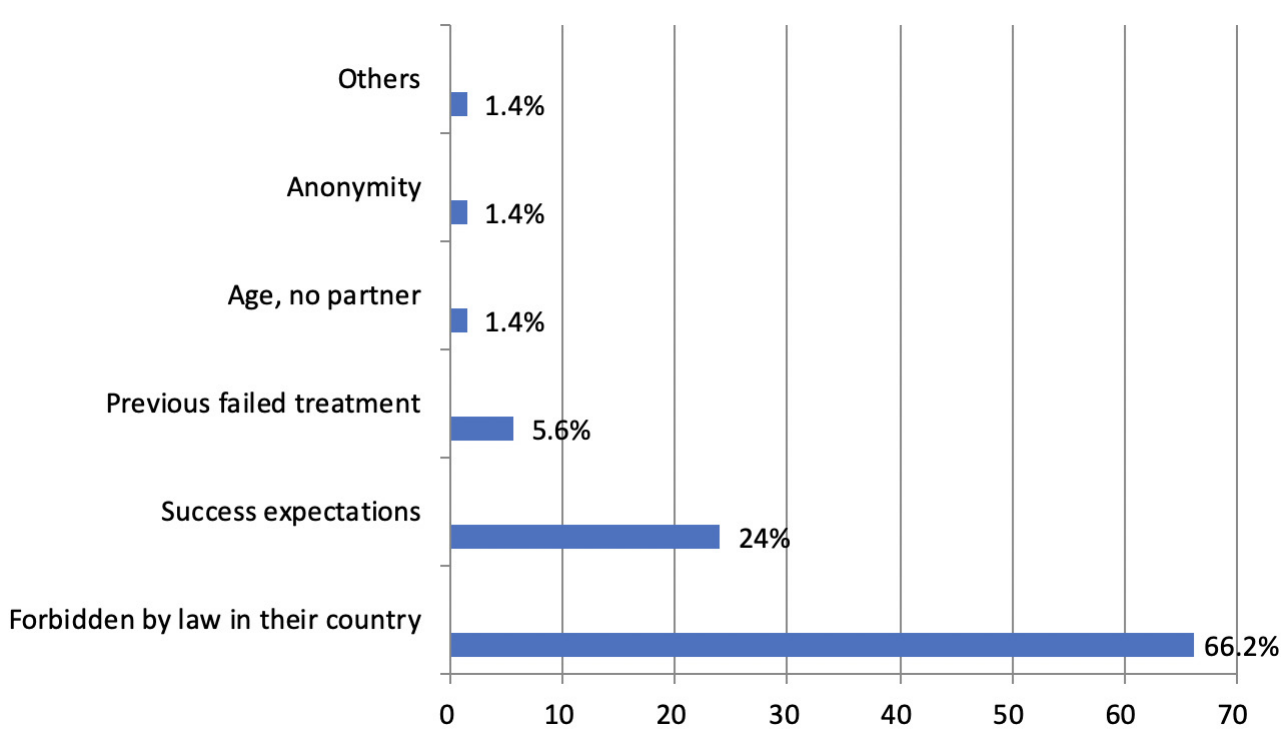
Mean reasons for patients choosing CBRC (Cross-border reproductive care).

**Fig 2:**
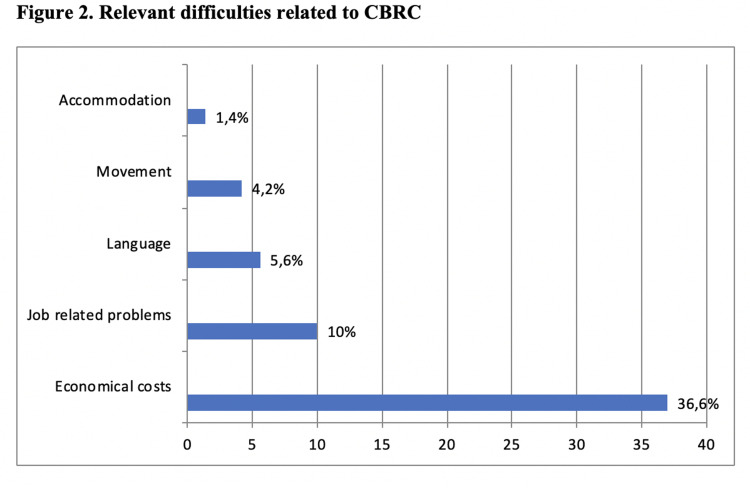
Main relevant difficulties related to CBRC (Cross-border reproductive care) referred by patients care) referred by patients

In the present study the women were accompanied by either their partners (89%), their partners and family/ friends (9.6%), or by only family/friends (1.4%). Frequency distribution of the main causes for CBRC are represented in Figure 1, which shows that the primary reason for foreign fertility treatment was the difficulty to access the desired treatment technique (66.2%). Generally, CBRC respondents felt supported by the international department (87.7%). The most positive aspects listed were linguistic-communication support (49.3%) and personalized monitoring (22%). Job-related problems and financial costs were the main relevant difficulties within the CBRC group (Fig .2). A total of 32.9% of CBRC respondents reported significant psychological discomfort, and most reported significant and relevant difficulties in cross-border infertility treatment (55.9%).

**Table 1:** Sample description


Sociodemographic and clinical variables		CBRC group (n=71)	Local group (n=90)	P value

Age (Y)		39.9 ± 5.08	38.8 ± 5.04	0.201
Origin				
	Spain	0 (0)	90 (100)	
	Italy	71 (100)	0 (0)	
Education level				
	Primary	9 (12.7)	11 (12.2)	0.835
	Secondary	29 (40.8)	33 (36.7)	
	University	33 (46.5)	46 (51.1)	
Civil status				
	Single	0 (0.00)	1 (1.10)	0.373
	Married-partner	71 (100)	89 (98.9)	
Partners’ gender				
	male	71 (100)	97 (96.7)	0.299
Employment status				
	Employed	65 (91.5)	78 (86.7)	0.052
	Duration of infertility (months)	48.8 ± 32.4	46.1 ± 39.6	0.657
	Previous infertility treatments: IUI	2.07 ± 2.47	1.77 ± 1.89	0.392
	Previous infertility treatments: IVFO	2.21 ± 2.75	1.03 ± 1.30	0.001^*^
	Previous infertility treatments: IVFD	0.21 ± 0.71	0.32 ± 0.85	0.378
	Recurrent pregnancy loss	26 (36.6)	24 (26.7)	0.143
Current treatment				
	IVFO	36 (50.7)	54 (60.0)	0.238
	IVFD	35 (49.3)	36 (40.0)	
	Personal psychiatric history	7 (9.9)	19 (21.1)	


Data are presented as mean ± SD or n (%) (n=161). SD; standard deviation, CBRC; Cross-border reproductive care, IUI; Intrauterine insemination, IVFO; *In vitro* fertilization with own oocytes, IVFD; In vitro fertilization with donated oocytes, and *; significant at P<0.05.

### Anxiety and depression levels of cross-border reproductive care women and local women

Table 2 shows the results obtained from analysis of variance (ANOVA) for comparing clinical scores between CBRC patients and local women, controlled for the IVF technique. CBRC women reported higher STAI-S scores, but this difference was only relevant (P<0.001) and statistically significant in receptor woman (IVF with donated oocytes). No differences in depression scores were found between CBRC women and local women.

### Personality profiles in cross-border reproductive care women and local women

Personality results are shown in Table 3 in a comparison of clinical scores between CBRC patients and local women, controlling for the IVF technique. In both groups, means in all subscales were within normal levels (+ 1 SD with regards to the general population) (21). No significant differences were found between groups, except for the Activity subscale with higher scores in the CBRC receptor group (IVF with donated oocytes; P=0.002).

**Table 2:** Comparison of STAI-S and BDI-II scores between the CBRC and local groups according to *in vitro* fertilization technique


Clinical assessment	CBRC group	n	Local grou	n	P value	MD (95% CI)

STAI-S IVFO	22.6 ± 9.70	36	20.8 ± 10.1	54	0.383	1.85 (-2.35; 6.06)
STAI-S IVFD	27.4 ± 6.85	35	18.8 ± 10.5	36	<0.000^*^	8.62 (4.42; 12.82)
BDI-II IVFO	8.06 ± 6.30	36	9.81 ± 7.15	54	0.234	-1.76 (-4.67; 1.16)
BDI-II IVFD	4.26 ± 4.04	35	5.36 ± 6.46	36	0.392	-1.10 (-3.66; 1.45)


Data are presented as mean ± SD or n (%); STAI-S; Spielberger State Anxiety Inventory, BDI-II; Beck Depression Inventory-II, IVFO; *In vitro* fertilization with own oocytes, IVFD; *In vitro* fertilization with donated oocytes, CBRC; Cross-border reproductive care, SD; Standard deviation, MD; Mean difference, CI; Mean difference confidence interval, *; significant at P<0.05.

**Table 3:** ZKPQ score comparison between the CBRC and local groups according to IVF technique


ZKPQ personality factors	CBRC group	n	Local group	n	P value	MD (95% CI)

Neuroticism-Anxiety IVFO	-0.44 ± 1.13	36	-0.33 ± 0.91	53	0.619	-0.109 (-0.54; 0.32)
Neuroticism-Anxiety IVFD	0.06 ± 0.74	35	0.04 ± 0.92	36	0.059	-0.435 (-0.88; 0.02)
Activity IVFO	0.16 ± 0.92	36	0.07 ± 1.10	53	0.702	0.086 (-0.36; 0.53)
Activity IVFD	0.45 ± 0.90	35	-0,21 ± 0.95	36	0.002*	0.711 (0.27; 1.15)
Impulsive Sensation Seeking IVFO	-0.27 ± 0.7	36	-0.66 ± 0.78	53	0.069	0.385 (0.06; 0.70)
Impulsive Sensation Seeking IVFD	-0.16 ± 0.84	35	-0.27 ± 1.11	36	0.624	0.115 (-0.35; 0.58)
Aggression-Hostility IVFO	-0.22 ± 0.74	36	-0.08 ± 1.02	53	0.452	-0.14 (-0.51; 0.23)
Aggression-Hostility IVFD	-0.08 ± 0.96	35	-0.01 ± 1.07	36	0.755	-0.076 (-0.56; 0.40)
Infrequency IVFO	0.63 ± 1.06	36	0.54 ± 1.22	53	0.725	0.08 (-0.41; 0.58)
Infrequency IVFD	1.26 ± 1.23	35	0.67 ± 1.60	36	0.086	0.592 (-0.08; 1.26)


Data are presented as mean ± SD or n (%); ZKPQ; Zuckerman-Kuhlman Personality Questionnaire, IVFO; *In vitro* fertilization with own oocytes, IVFD; *In vitro* fertilization with donated oocytes, SD; Standard deviation, CBRC; Cross-border reproductive care, *; Significant at P<0.05.

## Discussion

This study analysed whether there were psychopathological differences between CBRC women and local women undergoing IVF. We explored CBRC issues and the clinical, sociodemographic and personality profiles of both groups.

In a similar way to another Spanish study (24), the majority of women in the CBRC group were from Italy where Spain's CBRC treatment approach is particularly well-perceived (13). The main cause of cross-border travel in the CBRC group was the illegality/difficulty of access to the practice in their home country, which explained why all of the participants originated from Italy, a country with some of the most highly restrictive laws in Europe in terms of medically assisted procreation (25).

Both clinical groups showed a similar profile with respect to sociodemographic and clinical features. The only difference was that the CBRC group had previously undergone more ART, specifically more IVF with their own oocytes. This was in line with the causes of CBRC described by these patients in our study, most of whom had had experienced failed treatments and came to Spain to receive infertility treatment that was illegal in Italy.

Regarding psychopathology, our study finds higher anxiety-state levels in CBRC oocyte recipient patients in comparison with the local group. These findings suggest that anxiety is simultaneously associated with the migratory process and the type of ART used. These findings cannot be compared with other results as there have been no similar previous investigations. In terms of depression, no significant differences were found between the groups, which was in line with other research where women who underwent CBRC IVF in Spain had low levels of depression (24). Therefore, the present data has supported the position that the migratory process, when an oocyte donation is needed, exacerbates anxiety symptomatology. Previous studies described high and moderate levels of anxiety in women requiring donor oocytes, assessed immediately prior to the IVF (26). Before treatment, many oocyte recipients expressed concern about the lack of a genetic tie to a child born after the donor procedure and doubts about whether or not to disclose the donation (27). These fears could increase anxiety levels; thus, a psychological consultation prior to treatment with gamete donation is recommended (28). In addition, Italian women who undergo treatment with oocyte donation endure a technique that was illegal in Italy until 2013 (13, 29). Currently, despite being legal, oocyte donation presents with greater difficulties in terms of access compared to other countries, which potentially increases patients’ anxiety levels. All of the above factors add to the anxiety generated by infertility itself and support the observation that anxiety-related symptoms of infertile women are more prominent than those of fertile females (30). Furthermore, repeated fertility treatments and the accumulation of unsuccessful IVF treatments generate a sensation of vulnerability-based anxiety (31). The CBRC women in our study had more previous infertility treatments with their own oocytes. The psychological burden of conceiving through the donation of oocytes is added to the process of undergoing the treatment outside their country of origin. CBRC poses new challenges and difficulties for patients, such as having to live away from home and to adapt to an unknown country with possible language barriers and little social support (32). In fact, a third of the women in the CBRC group endorsed significant psychological significant discomfort, and more than half reported significant and relevant difficulties in cross-border infertility treatment. As reported in other studies (33), the majority of psychological discomfort in our CBRC sample arose from economical costs, with absence from the participant's workplace listed as the second most relevant difficulty.

In light of these results, assisted reproduction centres that assist CBRC patients should be prepared to identify patients’ anxiety levels prior to treatment, especially with oocyte donation and, if necessary, facilitate patient access to psychological support. Previous CBRC research in Spain (24) showed that couples with a history of oocyte donation treatments were more likely to perceive psychosocial support as useful and to desire it. Psychosocial interventions for couples under treatment for infertility, particularly cognitive behaviour therapy, has proven to be effective, both in reducing psychological distress and in improving clinical pregnancy rates.

On the other hand, no significantly different personality profiles were obtained between both groups, other than the Activity characteristic. This means that the CBRC group, specifically when an oocyte donation was required, was characterized by a greater tendency for general activity, an inability to relax and do nothing when the opportunity arises, a preference for hard and challenging work, a busy life, and a high energy level (34). CBRC patients who need oocyte donations tend to face more difficulties accessing reproductive treatment, thus making this personality tendency coherent in this subgroup of CBRC women given that CBRC recipient women must develop a proactive attitude towards infertility. These women must overcome the barriers and regulations of their countries to be able to carry out the reproduction treatment necessary for them to become mothers.

Finally, regarding CBRC issues and in accordance with previous studies (35), the main cause of reproductive tourism in our sample was the legal prohibition in the country of origin. This finding stresses the importance of taking a rapidly changing legal environment into consideration and to promote the adequate regulation of ART (36).

The present study is not without its limitations. First, all data were collected only from women who sought ART treatment. Future studies should aim to assess their partners in in order to obtain a more comprehensive view of CBRC effects (24). Second, the CBRC patients were from Italy, which limited the external validity of our analysis and comparisons with other countries of origin. The existence of country-based differences in the mental health of couples who undergo CBRC has been reported in previous studies (24), which suggests that this kind of analysis could be of interest. Third, the evaluation was carried out only through questionnaires, without a complementary clinical interview. Fourth, the cross-sectional perspective of this study did not permit the detection of differences between both groups after clinical intervention. It would be of interest to determine if a relationship existed between anxiety levels prior to fertility treatment and during pregnancy or postpartum. Finally, despite having been identified in infertile women (10), we did not assess coping strategies, cognitive style, quality of life or other psychopathologies of interest, such as somatic disorders.

## Conclusion

This study provides further information about the existence of increased anxiety in CBRC women, specifically those who receive oocyte donations. The findings suggest that screening systems and psychological support for anxiety in this population should be considered in order to improve the quality of care in CBRC.

## References

[1] Schmidt L (2009). Social and psychological consequences of infertility and assisted reproduction - what are the research priorities?. Hum Fertil (Camb).

[2] Ahuja KK (2015). Patient pressure: is the tide of cross-border reproductive care beginning to turn?. Reprod Biomed Online.

[3] Couture V, Drouin R, Tan SL, Moutquin JM, Bouffard C (2015). Crossborder reprogenetic services. Clin Genet.

[4] Sociedad Española de Fertilidad (2016). Informes Registro Nacional de Actividad-Registro SEF.Sociedad Española de Fertilidad.

[5] Fekkes M, Buitendijk SE, Verrips GH, Braat DD, Brewaeys AM, Dolfing JG (2003). Health-related quality of life in relation to gender and age in couples planning IVF treatment. Hum Reprod.

[6] Verhaak CM, Smeenk JM, Nahuis MJ, Kremer JA, Braat DD (2007). Longterm psychological adjustment to IVF/ICSI treatment in women. Hum Reprod.

[7] Chachamovich JR, Chachamovich E, Zachia S, Knauth D, Passos EP (2007). What variables predict generic and health-related quality of life in a sample of Brazilian women experiencing infertility?. Hum Reprod.

[8] Turner K, Reynolds-May MF, Zitek EM, Tisdale RL, Carlisle AB, Westphal LM (2013). Stress and anxiety scores in first and repeat IVF Cycles: a pilot study. PLoS One.

[9] Aarts JW, van Empel IW, Boivin J, Nelen WL, Kremer JA, Verhaak CM (2011). Relationship between quality of life and distress in infertility: a validation study of the Dutch FertiQoL. Hum Reprod.

[10] Cunha M, Galhardo A, Pinto-Gouveia J (2016). Experiential avoidance, self-compassion, self-judgment and coping styles in infertility. Sex Reprod Healthc.

[11] Lahey BB (2009). Public health significance of neuroticism. Am Psychol.

[12] Brown TA, Barlow DH (2009). A proposal for a dimensional classification system based on the shared features of the DSM-IV anxiety and mood disorders: implications for assessment and treatment. Psychol Assess.

[13] Zanini G (2011). Abandoned by the State, betrayed by the Church: Italian experiences of cross-border reproductive care. Reprod Biomed Online.

[14] Liu K, Case A, Cheung AP, Sierra S, AlAsiri S, Carranza-Mamane B (2011). Advanced reproductive age and fertility. J Obstet Gynaecol Can.

[15] Chambers GM, Sullivan EA, Ishihara O, Chapman MG, Adamson GD (2009). The economic impact of assisted reproductive technology: a review of selected developed countries. Fertil Steril.

[16] Pennings G, De Wert G, Shenfield F, Cohen J, Tarlatzis B, Devroey P (2008). ESHRE task force on ethics and law 15: cross-border reproductive care. Hum Reprod.

[17] Millbank J (2015). Responsive regulation of cross-border assisted reproduction. J Law Med.

[18] Spielberger CD, Gorsuch RL, Lushene RE (2008). STAI.Cuestionario de ansiedad estado-rasgo.

[19] Beck AT, Ward CH, Mendelson M, Mock J, Erbaugh J (1961). An inventory for measuring depression. Arch Gen Psychiatry.

[20] Beck AT, Steer RA, Brown GK, Ghisi M, Flebus GB, Montano A, Sanavio E, Sica C (2007). BDI-II Manual. Firenze: Giunti OS Organizzazioni Speciali; 2007; 1-79.

[21] Zuckerman M, Kuhlman DM, Joireman J, Teta P (1993). A comparison of three structural models for personality: the big three, the big five, and the alternative five. J Pers Soc Psychol.

[22] Gomà-i-Freixanet M, Valero Ventura S (2008). Spanish normative data of the Zuckerman-Kuhlman Personality Questionnaire in a general population sample. Psicothema.

[23] De Pascalis V, Russo PM (2003). Zuckerman-Kuhlman Personality Questionnaire: preliminary results of the Italian version. Psychol Rep.

[24] Madero S, Gameiro S, García D, Cirera D, Vassena R, Rodríguez A (2017). Quality of life, anxiety and depression of German, Italian and French couples undergoing cross-border oocyte donation in Spain. Hum Reprod.

[25] Boggio A (2005). Italy enacts new law on medically assisted reproduction. Hum Reprod.

[26] Lisovskaya TV, Zakhezina EA, Filippova GG, Ambartsumyan EM, Portnov IG, Mayasina EN (2017). Mental state assessment of recipients in the IVF donor programs and psychotherapeutic methods of its correction. Gynecol Endocrinol.

[27] Hammarberg K, Carmichael M, Tinney L, Mulder A (2008). Gamete donors’ and recipients’ evaluation of donor counselling: a prospective longitudinal cohort study. Aust N Z J Obstet Gynaecol.

[28] Boivin J, Griffiths E, Venetis CA (2011). Emotional distress in infertile women and failure of assisted reproductive technologies: metaanalysis of prospective psychosocial studies. BMJ.

[29] Shenfield F, de Mouzon J, Pennings G, Ferraretti AP, Andersen AN, de Wert G (2010). Cross border reproductive care in six European countries. Hum Reprod.

[30] Lakatos E, Szigeti JF, Ujma PP, Sexty R, Balog P (2017). Anxiety and depression among infertile women: a cross-sectional survey from Hungary. BMC Womens Health.

[31] Greil AL, McQuillan J, Lowry M, Shreffler KM (2011). Infertility treatment and fertility-specific distress: A longitudinal analysis of a populationbased sample of U.S.women. Soc Sci Med.

[32] Blyth E, Thorn P, Wischmann T (2011). CBRC and psychosocial counselling: assessing needs and developing an ethical framework for practice. Reprod Biomed Online.

[33] Blyth E (2010). Fertility patients’ experiences of cross-border reproductive care. Fertil Steril.

[34] Gomà-i-Freixanet M, Valero S, Muro A, Albiol S (2008). Zuckerman-Kuhlman Personality Questionnaire: psychometric properties in a sample of the general population. Psychol Rep.

[35] Van Hoof W, Provoost V, Pennings G (2013). Reflections of Dutch patients on IVF treatment in Belgium: a qualitative analysis of internet forums. Hum Reprod.

[36] Jackson E, Millbank J, Karpin I, Stuhmcke A (2017). Learning from crossborder reproduction. Med Law Rev.

